# Prediction of a Therapeutic Dose for Buagafuran, a Potent Anxiolytic Agent by Physiologically Based Pharmacokinetic/Pharmacodynamic Modeling Starting from Pharmacokinetics in Rats and Human

**DOI:** 10.3389/fphar.2017.00683

**Published:** 2017-10-10

**Authors:** Fen Yang, Baolian Wang, Zhihao Liu, Xuejun Xia, Weijun Wang, Dali Yin, Li Sheng, Yan Li

**Affiliations:** ^1^Key Laboratory of Carcinogenesis and Translational Research (Ministry of Education), Center of Drug Clinical Trial, Peking University Cancer Hospital and Institute, Beijing, China; ^2^Clinical Pharmacology Research Center, Peking Union Medical College Hospital and Chinese Academy of Medical Sciences, Beijing, China; ^3^Department of Drug Metabolism, Beijing Key Laboratory of Non-Clinical Drug Metabolism and PK/PD Study, Institute of Materia Medica, Chinese Academy of Medical Sciences and Peking Union Medical College, Beijing, China; ^4^Department of Drug Delivery System, Institute of Materia Medica, Chinese Academy of Medical Sciences and Peking Union Medical College, Beijing, China; ^5^Department of Pharmacology, Institute of Materia Medica, Chinese Academy of Medical Sciences and Peking Union Medical College, Beijing, China; ^6^Department of Synthetic Medicinal Chemistry, Institute of Materia Medica, Chinese Academy of Medical Sciences and Peking Union Medical College, Beijing, China

**Keywords:** buagafuran, pharmacokinetics, pharmacodynamic, physiologically based pharmacokinetic (PBPK) modeling, *in silico* modeling, clinical pharmacokinetics

## Abstract

Physiologically based pharmacokinetic (PBPK)/pharmacodynamic (PD) models can contribute to animal-to-human extrapolation and therapeutic dose predictions. Buagafuran is a novel anxiolytic agent and phase I clinical trials of buagafuran have been completed. In this paper, a potentially effective dose for buagafuran of 30 mg t.i.d. in human was estimated based on the human brain concentration predicted by a PBPK/PD modeling. The software GastroPlus^TM^ was used to build the PBPK/PD model for buagafuran in rat which related the brain tissue concentrations of buagafuran and the times of animals entering the open arms in the pharmacological model of elevated plus-maze. Buagafuran concentrations in human plasma were fitted and brain tissue concentrations were predicted by using a human PBPK model in which the predicted plasma profiles were in good agreement with observations. The results provided supportive data for the rational use of buagafuran in clinic.

## Introduction

Buagafuran is a derivative of agarofuran which was extracted from *Aquilaria agallocha* Roxb. Buagafuran exhibits significant activity in several animal models of anxiety, such as rat and mouse EPM test, rat social interaction test and the safety signal withdrawal test (Li et al., 2003, 2013; Liu et al., 2003). In addition to the modulation of central monoamine neurotransmitters (Zhang et al., 2004), the anxiolytic effects of buagafuran may also have relation with the inhibition of neuronal delayed rectifier potassium channels (Chen C.L. et al., 2013). In the pharmacokinetic (PK) study, buagafuran was shown to undergo extensive metabolism. Although the total radioactivity recovered was about 80% (51.2% in urine and 28.7% in feces) in rats after oral administration of buagafuran, less than 0.1 and 0.3% of the dose are recovered as prototype in the urine and bile, respectively (Hu et al., 2011). Similar extraction ratio of buagafuran (about 50%) and metabolite profiles were found in rat and human liver microsomes. It was found that CYP3A and CYP2E were the major enzymes involved in its metabolism *in vitro* (Zhang, 1997; Li et al., 2001; Li, 2010). In phase I clinical trials, buagafuran was proved to be well tolerated in healthy volunteers at multiple doses up to 120 mg twice daily (Wang et al., 2011; Chen X. et al., 2013; Yang et al., 2013). Before further study, it is very important to clear the dose exposure–response relationship for buagafuran on the target which could facilitate the design of clinical pharmacology trial.

Physiologically based pharmacokinetic (PBPK) models are mathematical models that combine anatomical and physiological properties of animals or humans, physicochemical parameters of compounds, and formulation parameters of drug candidates for predicting the absorption, distribution, metabolism and excretion of drugs in clinic (Shardlow et al., 2013). Based on PBPK model, PBPK-pharmacodynamic (PBPK/PD) model was usually developed to provide the essential dose–response relationships. It has been recognized as a powerful tool to extrapolate PK or dose across different species (De Buck et al., 2007; Allan et al., 2008; Brochot et al., 2010; Bungay et al., 2011; Yamazaki et al., 2011; Weber et al., 2012; Wang et al., 2015). Besides, it was thought that PBPK/PD modeling was assisted in defining mechanism of action and optimizing dosage regimens.

In this study, a PBPK/PD modeling prediction for buagafuran was constructed. On the basis of the prediction of brain PK profile of buagafuran in human, the potentially effective doses of buagafuran in human were predicted.

## Materials and Methods

### Material

**FIGURE 1:**
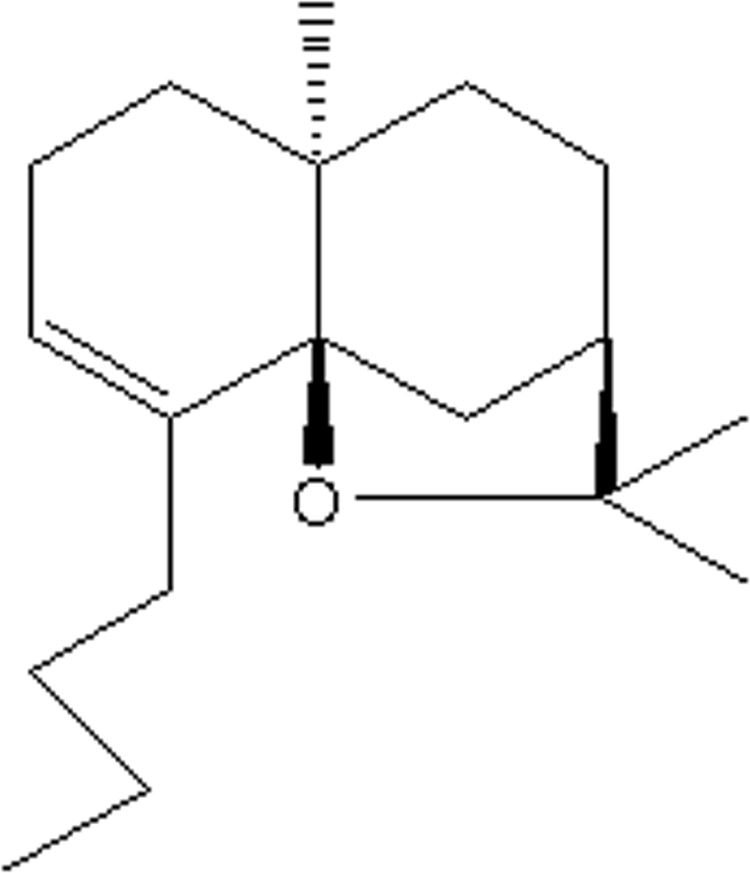
The chemical structure of buagafuran.

Buagafuran (chemical purity > 99%) and ^3^H-buagafuran were provided by Department of Synthetic Medicinal Chemistry (Chinese Academy of Medical Sciences, Beijing, China). The chemical structure of buagafuran was shown in **Figure 1**. ^3^H-buagafuran had a specific activity of 12.5 mCi/mmol with radiochemical purity > 95%. Buagafuran formulation of PVP (Xia et al., 2008) was provided by Department of Synthetic Medicinal Chemistry and Department of Drug Delivery System (Chinese Academy of Medical Sciences, Beijing, China). Halofantrine served as the IS (purity > 98%) and purchased from Sigma (St. Louis, MO, United States). All other chemicals were of analytical grade.

### PK in the Rat

Animal Care and Welfare Committee of Institute of Materia Medica, Chinese Academy of Medical Sciences approved all animal protocols (1 Xian nong tan Street, Xicheng District, Beijing, China; Permission Number: SYXK 2014-0023, Expiration: 06 Aug, 2019). All animal programs are in compliance with the Guide for the Care and Use of Laboratory Animals issued by Beijing Association on Laboratory Animal Care (BALAC). Buagafuran formulation of PVP was suspended in deionized water and was orally administered at 4 or 8 mg/kg to male Wistar rats (weighing 180–200 g). Brain samples treated with the dose of 4 mg/kg and blood samples of both treatments were collected over 3 h. Plasma samples were obtained by centrifugation, and brain tissues were homogenized in a threefold volume (w/v) of saline. Buagafuran concentrations in plasma and brain samples were quantitated by an LC-MS/MS method (Yang et al., 2011).

A volume of 200 μL plasma or tissue homogenate was mixed with 100 μL IS (10 ng/mL) followed by addition of 2 mL hexane. After vortexing for 2 min, the mixture was centrifuged for 5 min at 3000 *g*. The extracted solution was combined and evaporated to dryness at 25°C. The residues were reconstituted in 80 μL of 80% methanol. The chromatographic separation and mass spectrometric analysis was performed on a LC/TSQ Quantum Access mass spectrometer (Thermo Finnigan, San Jose, CA, United States). Ten microliter of the sample was injected onto an Agilent Eclipse Plus C_18_ analytical column (3.5 μm, 50 mm × 2.1 mm) and then buagafuran and IS were eluted with a gradient of acetonitrile (phase B) and water containing 0.1% v/v formic acid (phase A). The elution program was applied as follows: 50% B for 0–0.5 min, 50% B→97% B for 0.6–5.5 min, 50% B for 5.6–7.6 min. The outlet of the column was detected in positive ionization electrospray mode with selected reaction monitoring. The monitored transitions of buagafuran and IS were m/z 245→105 and 500→142, respectively. The retention time (RT) of buagafuran was 6.13 min. The linear range of the calibration curve was 0.5–800 ng/mL.

### Clinical PK

The study protocol was approved by the Ethics Committee of Peking Union Medical College Hospital approved (Permission Number: 09103), and the Informed Consent Form was signed by all subjects before the study. PVP formulation of buagafuran was packed into capsules for oral administration. In a study of open-label, randomized, cross-over and dose-ranging design, 12 healthy male Chinese volunteers (aged 22–40 with weighing 55–70 kg) received a single oral dose of buagafuran (30, 60, and 120 mg). Serial blood samples were collected up to 48 h. In a phase I, randomized and multiple oral dose study, 5 healthy male Chinese volunteers (aged 20–31 with weighing 55–73 kg) received once-daily oral dose of 60 mg buagafuran for 7 days. On day 1 and 7, plasma samples were collected at the designed time points. Detailed clinical data was previously reported (Yang et al., 2011, 2013).

### PK Analysis

Pharmacokinetic parameters were determined with a non-compartmental model using WinNonlin v 6.0 (Pharsight Corporation, Mountain View, CA, United States). The area under the compound concentration versus time curve from time zero to the last time point (AUC_0-t_) and extrapolated to infinity (AUC_0-∞_) and the peak plasma concentration (*C*_max_) were calculated.

### Clearance (CL) Prediction from Incubation with Liver Microsomes

Drug disappearance *t*_1/2_ value measurements in rat and human microsomes were used to determine the *in vitro* apparent intrinsic clearance value (*CL*_intapp_) of buagafuran acquired using Then *CL*_intapp_ was converted to *in vivo* intrinsic clearance (*CL*_int_).

Rat or human liver microsomal incubation (0.5 mg/mL) was carried out at 37°C in a mixture of 0.5 mL in 0.05 M Tris–HCl buffer (pH 7.4). Buagafuran (2 μM) was added to the incubation system. The addition of NADPH (1 mM) initiated the reaction, then the reaction was incubated for 0, 10, 20, 30, 45, and 60 min. The equal volume of acetonitrile containing the IS (10 ng/mL) was added to stop the reaction. The mixtures were centrifuged at 16000 *g* for 5 min and the supernatants were determined using LC-MS/MS method.

The natural log (In) of buagafuran concentration versus time was plotted and the slope of the best fit line of was determined by the data (*k*). This was converted into *in vitro t*_1/2_ where *t*_1/2_ = In 2/*k*. *CL*_intapp_ was calculated by the following equation:

(1) $$\begin{array}{c} CL_{\mathrm{int}\left(\mathrm{app}\right)}=\left(\frac{k}{M}\right) \end{array}$$

Where *M* is the microsomal protein concentration in the incubation. *CL*_int_ by liver was calculated from equation 2:

(2) $$\begin{array}{c} CL_{\mathrm{int}}=CL_{\mathrm{int}\left(\mathrm{app}\right)}\times \frac{\mathrm{mgmicrosome}}{\mathrm{gliver}}\times \frac{\mathrm{liverweight}\left(g\right)}{\mathrm{bodyweight}\left(\mathrm{kg}\right)} \end{array}$$

Where a value of 45 mg of protein per gram of liver tissue was used for both rat and human, and a liver weight of 40 and 21 g per kg were applied to rat and human, respectively (Hosea et al., 2009). For calculation of *in vivo* hepatic clearance, Well-stirred model is usually applied (De Buck et al., 2007; Zientek et al., 2010; Liu et al., 2014). The calculation equation is as follows:

(3) $$\begin{array}{c} CL_{\mathrm{hep},\mathrm{blood}}\;=\;\frac{\left(Fu_{p}/R_{b}\right)\times Q_{h}\times \left(CL_{\mathrm{int}}/Fu_{\mathrm{inc}}\right)}{Q_{h}+{\left(CL_{\mathrm{int}}/Fu_{\mathrm{inc}}\right)}^{*}\left(Fu_{p}\;/R_{b}\right)} \end{array}$$

Where *Q*_h_ (rat, 70; human, 20 mL/min/kg) is the hepatic blood flow; *F*u_p_ is unbound fraction in plasma; *F*u_inc_ is unbound fraction in microsomal or hepatocyte incubation; *R*_b_ is the ratio of blood to plasma concentrations.

### Caco-2 Transport Studies

Caco-2 Cells from passage 38 were applied to the transport experiments. Cells were seeded on polycarbonate filters (12 mm diameter, pore size 0.4 μm). The cell monolayers were used 21 days post seeding. Buagafuran was dissolved in DMSO. It was further diluted to 50 μM with HBSS. The final buagafuran concentration was 50 μM. For absorptive (AP→BL) permeability test, drug solution (0.4 mL) was added to the apical chamber. For secretive (BL→AP) permeability experiments, drug solution (0.6 mL) was added to the basolateral chamber, then 50 μL samples (*n* = 3) were withdrawn from receiver chamber at specified times and 50 μL fresh buffer was added in the receiver chamber. The experiments were carried out in triplicate at 4 time points.

Transport of buagafuran was tested using a linear model. The regression line was validated by inspection with naked eye and the value of correlation coefficient was *R* > 0.9. The apparent permeability coefficient (*P*_app_) was calculated by the formula P_app_ = (ΔQ/Δt)/AC_o_, where ΔQ/Δt is the transport rate (nmol/s), which remained approximately constant in time, A is the well membrane area (0.6 cm^2^), and *C*_o_ is the initial concentration of buagafuran (μM). The efflux rate (ER) was the ratio of the *P*_app_ from the basolateral to apical cells versus the *P*_app_ from the apical to basolateral cells. Transport of the tested drug is linear over the incubation times of 30–120 min.

### Plasma Protein Binding

The plasma protein binding of buagafuran (0.5–2 μg/mL) was investigated by equilibrium dialysis. Aliquots (0.5 mL) of plasma were dialyzed against 10 mL phosphate buffered saline containing a ^3^H-buagafuran tracer over a membrane with an 8,000- to 12,000-dalton molecular weight cutoff. Dialysis experiment was performed for 60 h at 4 °C. After the dialysis period, 100 μL of the PBS fraction (containing unbound buagafuran) and 100 μL of the plasma fraction (containing both bound and unbound drug) was taken for liquid scintillation counting. The ratio of buagafuran concentration in buffer to that in plasma was applied to calculate the unbound fraction (Fu).

### PD in the Rat

Anxiolytic activity of buagafuran was evaluated by EPM model. The rat EPM consisted of two open arms (50 cm × 10 cm) and two closed arms (50 cm × 10 cm × 40 cm) extending from the central platform (10 cm × 10 cm), the maze rising from the floor by 50 cm.

Male Wistar rats, weighing between 130 and 160 g, were handled daily for 1 week prior to the study and were fasted for 12 h before experiment. Group I: the treated rats received buagafuran formulation of PVP (2, 4, and 8 mg/kg) via the oral route, while the rats that served as control group received vehicle (PVP). Experiments were carried out 10 min after oral administration of drugs or vehicles. Group II: Rats were evaluated 5, 10, 15, 30, 60, 90, 120, and 180 min after oral treatment with buagafuran (4 mg/kg). During a typical test, place each rat on the center of the maze, facing an open arm, and allowing free exploration of the maze for a 5 min test period. An entry was defined by all four paws enter an arm. The total number of arm entries, and the time spent in each type of arm was recorded. The maze was wiped down with 10% ethanol between tests. All experiments were carried out between 9:00 am and 12:00 am.

The difference between control and treatment group was statistically analyzed by one-way analyses of variance (ANOVA). Differences with probabilities of less than 5% (*p* < 0.05) were regarded as significant.

### PBPK/PD Modeling

GastroPlus^TM^ software (version 8.5) was used to perform all simulations, (Simulations Plus, Inc., Lancaster, CA, United States). The absorption of oral formulations from the GI tract was modeled by the advanced compartmental absorption and transit (ACAT^TM^) model implemented in GastroPlus (Agoram et al., 2001; Kuentz et al., 2006; Huang et al., 2009; Zhang et al., 2011). Input parameters in the ACAT model include three groups: formulation properties, physicochemical properties and pharmacokinetic parameters. The values of the first two groups for simulations of buagafuran were exhibited in **Table 1**. The immediate release (IR) suspension for rat and IR capsule for human were selected for simulation of buagafuran in GastroPlus^TM^. The ACAT model consists of nine compartments that mimic the GI tract. Each GI tract has an associated ASF. ASF is the multiplier used to scale the permeability to account for different surface-to-volume ratios, pH effects, transporter differences, and other factors that affect absorption between different compartments (Zhang et al., 2011). Four coefficients C1–C4 are used to calculate ASF. And there are eight basic ASF models in version 8.5. The Opt logD Model SA/V of the ASF models was applied to simulate the oral absorption of buagafuran in rat and human, taking into account the increased surface area associated with villi and microvilli.

**Table 1:** Input parameters used in the PBPK model for simulations of buagafuran.

Parameters	Values
MW	262.44
logP*^b^*	6.24
Solubility (mg/mL)*^b^*	0.00985
Particle density (g/mL)*^b^*	1.2
Mean precipitation time (s)*^b^*	900
Diffusion coefficient (cm^2^/s)*^b^*	0.8251
Drug particle density (g/mL)*^b^*	1.2
Mean particle radius (μm)*^b^*	5
Caco-2 Papp (cm^2^/s)*^a^*	19.5 × 10^-5^
Blood/plasma concentration ratio*^b^*	0.73
F_uphuman_ (%)*^a^*	3.1
F_uprat_ (%)*^a^*	4

*All predicted parameters were calculated by GastroPlus^*TM*^, version 8.5 (Simulations Plus, Inc., Lancaster, CA, United States); *^a^*measured values; *^b^*predicted values.*

The ‘optimization’ module helps to fit the ACAT models with the data obtained after oral dosing of buagafuran. Two coefficients (C1 and C2) used for calculation of ASFs in the physiology model were selected for optimization. Coefficient C3 and C4, applied to determine the colon ASFs, were GastroPlus^TM^ default values considering buagafuran is almost completely absorbed before it travels into the colon. The stomach transit time of suspension and capsule was 0.1 and 1 h, respectively. Other parameters were fixed at default values in GastroPlus^TM^.

The pharmacokinetic parameters of unbound fraction in plasma (F_Up_) were experimentally measured values. The blood/plasma ratio was *in silico* predictions. The *CL*_hep_ values from microsomal assay were used for baseline simulation of buagafuran in rat and human. According to the clinical data of buagafuran urine excretion less than 1% of the dose administered, the renal clearances of buagafuran in both rat and human in PBPK models were set to 0 (Chen X. et al., 2013).

The tissue to plasma partition coefficient (*K*p) was used to calculate the drug concentration in the extracellular compartment. Perfusion-restricted tissue has six models to simulate the *K*p values. Poulin and Theil-Homogeneous model was applied to calculate the *K*p in all simulations. All tissue *K*p values (except brain) were *in silico* predictions. *K*p brain was optimized by parameter sensitivity analysis (PSA).

Parameter sensitivity analysis was carried out for parameters with uncertainty, including coefficients (C1, C2, C3, and C4) in ASFs calculation, diffusion coefficient, system clearance and *K*p brain. When PSA is executed, a parameter changed gradually while fixing the other parameters at the baseline level.

The PD Plus module implemented in GastroPlus was used. Based on the parameters (*R*^2^ and Akaike criterion) in PK-PD model simulation, the best-fitting model, Sigmoid *E*_max_ model, was employed to fit therapeutic PD response (time spent on the open arms) with the brain concentration profile of buagafuran in rat using the equation 4:

(4) $$\begin{array}{c} E\;=\;E_{0}+\;\frac{E_{\max}C^{\gamma }}{EC_{50}^{\;\gamma }+C^{\gamma }}. \end{array}$$

where *C* is in ng/mL, *E*_0_ is the time spent by the control group in the open arms which is equal to 0.63, *E*_max_ is the maximum response, *EC*_50_ is the concentration of a compound at which 50% of its maximum response is observed and γ is the Hill coefficient. The set of parameter values for the model was determined by GastroPlus software. GastroPlus simulation was then used to predict the brain concentration profile and PD response profile of buagafuran in human.

## Results

### PBPK Modeling of Buagafuran in Rats and Humans

Parameters in **Table 1** were used for simulation of plasma PK profiles in rat and human after oral administration of buagafuran. The apparent permeability coefficients of buagafuran transport were (19.5 ± 4.6) × 10^-5^ cm/s (AP to BL) and (43.8 ± 5.3) × 10^-5^ cm/s (BL to AP). In rat and human plasma, there was no evident concentration-dependent binding of buagafuran with plasma protein in the range of 0.5–2 μg/mL. Consequently, a single Fu value was applied to rat and human based on the overall average of the data in the entire concentration range. The unbound fraction of buagafuran was 3.1% in rat plasma and 4.0% in human plasma.

After optimization of the ‘optimization’ module, the ASF models were further validated by comparing the simulation with the observed data. The constant C1 of Opt logD Model SA/V was adjusted to be 0.6805 in rat and 0.8005 in human. The constants C2 in both rat and human models remained at its default value. The optimized ASF values of rat were similar to those of human, indicating a similar absorption of buagafuran between the two species.

With the purpose of making a buagafuran PBPK simulation, it is essential to estimate *K*p values, which are estimated using Poulin and Theil equation and Rodgers and Rowland equation. The main difference between the above two equations is the first equation considers drug ionization and specific interactions between bases and acidic tissue phospholipids. Therefore, the Poulin and Theil-Homogeneous model was used for *K*p calculation of buagafuran in rat and human because of buagafuran being a neutral drug. The brain *K*p in rat was optimized to 6.7 and was used for the simulation of buagafuran in human. Default values in GastroPlus software were applied for *K*p of other organs in rat or human.

Clearance is a key parameter for PBPK simulation. The CL of buagafuran was predicted using rat and human liver microsomes. The *in vitro CL*_intapp_ (result from Equation 1) was 0.033 mL/min/mg for rat and 0.022 mL/min/mg for human. *CL*_hep_ values of buagafuran predicted were 28.0 mL/min/kg in rat liver microsomes and 7.7 mL/min/kg in human liver microsomes. By comparing the fitting data with the observed data for further optimization, the input *CL*_hep_ values were 36.8 mL/min/kg for rat and 11.7 mL/min/kg for human. The results showed that buagafuran has moderate hepatic clearance in both rats and humans.

### Prediction of Plasma or Brain PK in Rat and Human

**FIGURE 2:**
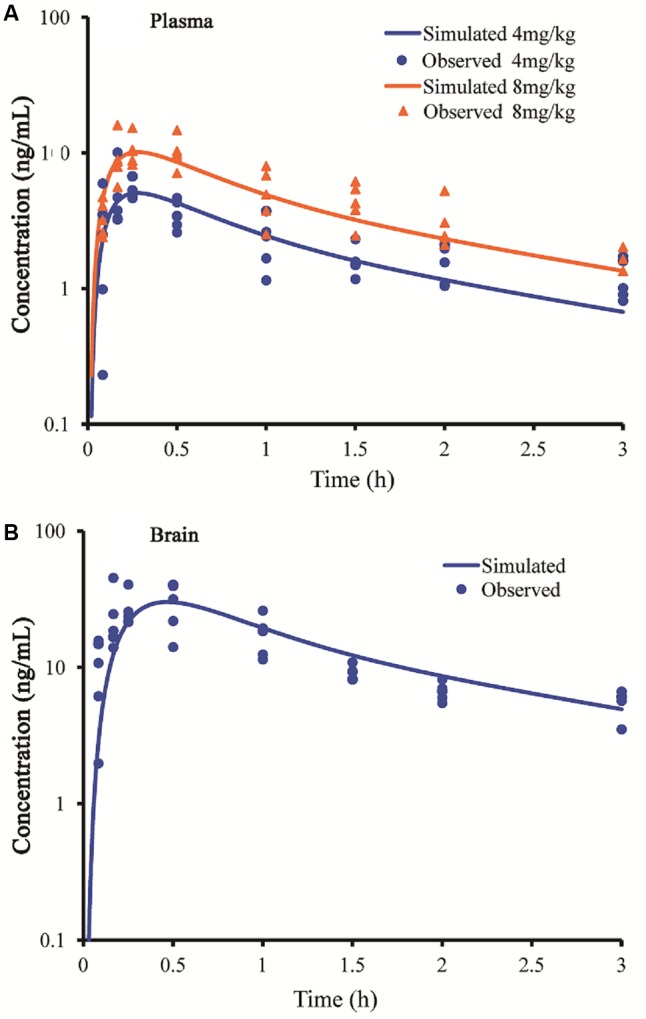
Simulated and observed plasma **(A)** and brain **(B)** concentration-time profiles of buagafuran in rats after a single oral dose of 4 mg/kg (circles) and 8 mg/kg (triangles). Circles and triangles correspond to independent observations in rats (*n* = 5). Solid line represent the prediction for buagafuran.

After the models were adjusted, simulated and observed plasma concentration-time profiles of buagafuran in rat after single oral dosing (4 and 8 mg/kg) were in good agreement, as shown in **Figure 2A** and **Table 2**. After oral administration, the plasma concentrations of buagafuran increased rapidly and the plasma concentration reached a peak at 15 min after administration.

**Table 2:** Observed and simulated mean plasma PK parameters of buagafuran.

Species	Oral dosing	*T*_max_ (h)		*C*_max_ (ng/mL)		MRT_(0-∞)_ (h)		AUC_0-t_ (ng⋅h/mL)		AUC_0-∞_ (ng⋅h/mL)	
		Observed	Simulated	Observed	Simulated	Observed	Simulated	Observed	Simulated	Observed	Simulated
Rat	4 mg/kg	0.25	0.28	5.31	5.06	3.54	1.68	6.69	6.14	11.43	7.44
	8 mg/kg	0.25	0.28	10.64	10.13	1.21	1.68	14.15	12.28	15.06	14.87
Human	Single dosing/30 mg	1.00	1.12	19.47	19.73	3.33	8.43	47.62	50.13	54.91	55.79
	Single dosing/60 mg	1.00	1.12	39.77	39.55	4.87	8.42	92.40	100.24	95.38	111.54
	Single dosing/120 mg	1.00	1.20	81.64	76.20	5.28	8.50	246.48	200.43	271.68	222.99
	Multiple dosing/first dosing	2.00	1.20	27.08	31.59	9.94	7.15	99.40	104.19	113.48	111.65
	Multiple dosing/last dosing	3.00	1.20	32.00	32.58	20.89	13.58	163.30	124.56	230.74	152.98

*Observed PK parameters were calculated from plasma drug concentration-time profiles by WinNonlin v6.0 (Pharsight Corporation, Mountain View, CA, United States), while simulated ones were performed using GastroPlus^*TM*^ software, version 8.5 (Simulations Plus, Inc., Lancaster, CA, United States).*

The simulated brain concentration-time profile after oral administration of buagafuran to rat was compared with the observed data (**Figure 2B**). Brain penetration was rapid, and the concentrations were detected at 5 min and the maximum concentrations in brain were obtained at 15–30 min after oral administration.

**FIGURE 3:**
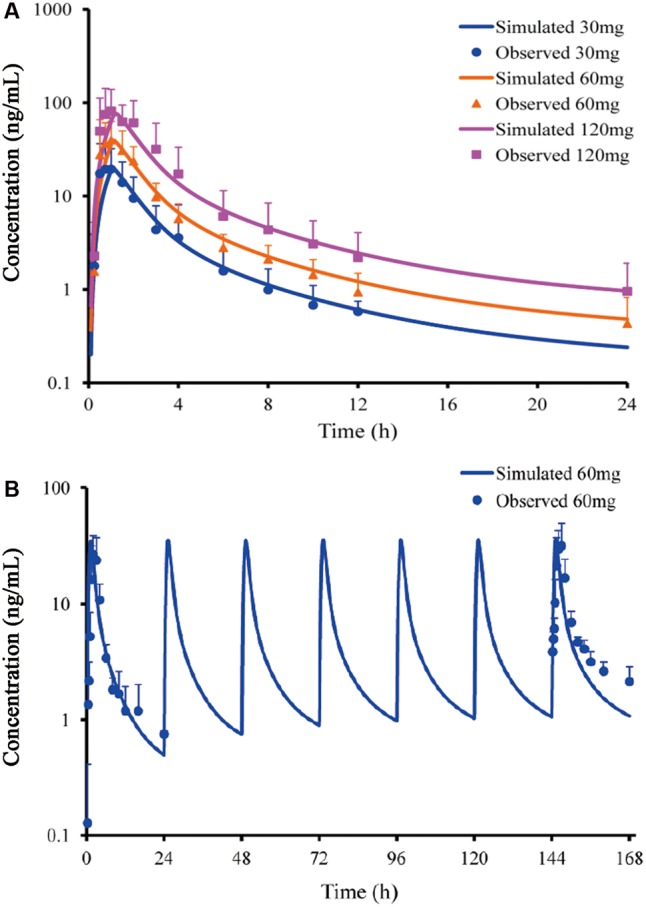
Simulated and observed plasma concentration-time profiles of buagafuran in human after single oral doses **(A)** of 30 (circles), 60 (triangles) and 120 mg (squares) (*n* = 12) and multiple oral 60 mg dosing (**B**, *n* = 5). Circles, triangles and squares correspond to mean observation; each point represents the mean ± SD. Solid lines represent the prediction for buagafuran.

Predicted PK profiles were close to the PK curves of buagafuran observed in human with the dose range of 30–120 mg (**Figure 3A** and **Table 2**). Buagafuran absorption was rapid after oral dosing with time to peak plasma concentration (*T*_max_) ranging from 0.5 to 2 h. *T*_max_ was similar across doses. The multiple-dose PK of buagafuran (60 mg) in healthy men was simulated well (**Figure 3B**).

When the PSA was performed in the simulation, we found that the PK parameters, AUC and *C*_max_, were highly affected by the coefficient C1 in ASF model. Finally, the coefficient was optimized to 0.6805 for rat and 0.8005 for human.

### PD in the EPM Model

**FIGURE 4:**
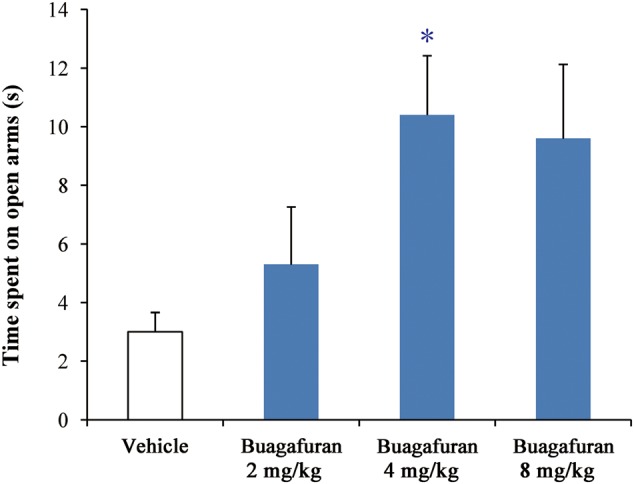
The effects of buagafuran (2, 4, and 8 mg/kg) administered orally 5 min prior to 5-min test on the time spent on the open arms in the elevated plus-maze test. Each column represents the mean ± SEM. (*n* = 10) ^∗^*p* < 0.05 vs. vehicle.

As shown in **Figure 4**, buagafuran significantly prolonged the total time spent in open arms after oral administration. The effect was dose-dependent. After oral administration at a dose of 4 mg/kg, buagafuran exerted its maximum effect (*p* < 0.05 versus vehicle), and the action slightly decreased at a higher dose (8 mg/kg), indicating an inverted U-shaped dose response curve.

**FIGURE 5:**
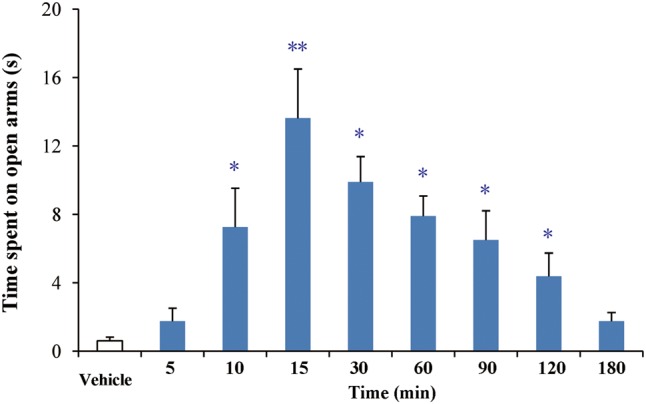
The effects of buagafuran (4 mg/kg) on the time spent on the open arms in the elevated plus-maze test. Each column represents the mean ± SEM. (*n* = 10) ^∗^*p* < 0.05, ^∗∗^*p* < 0.01 vs. vehicle.

**FIGURE 6:**
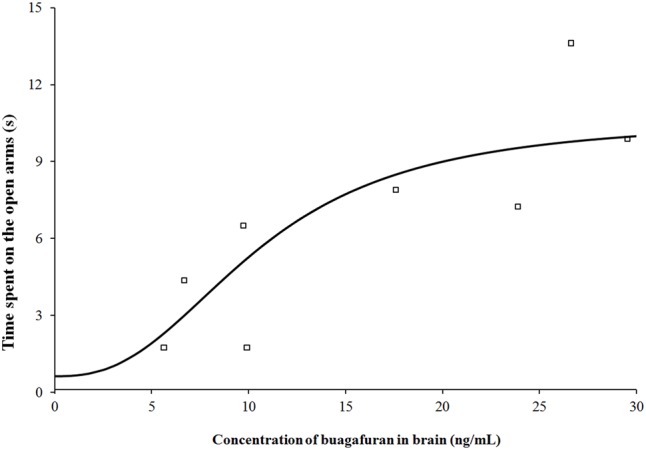
Pharmacodynamic response (time spent on the open arms) with brain concentration profile of buagafuran in rats. The open square symbols correspond to the mean observed values. The solid line represents the prediction for buagafuran.

After oral administration of 4 mg/kg, buagafuran has a rapid onset of action (within 10 min, *p* < 0.05 versus vehicle) and reached the maximal level of activity at 15 min (*p* < 0.01 versus vehicle). Its activity decreased within 2 h (*p* < 0.05 versus vehicle) after dosing (**Figure 5**). The therapeutic PD response (time spent in the open arms) with the brain concentration profile of buagafuran was described by a direct sigmoidal *E*_max_ model (**Figure 6**). No effect delays between the response and effects were found. *EC*_50_ was 10.6 ng/mL and Hill coefficient was 2.5.

### Human Dose Prediction on the Basis of PBPK/PD Modeling

**FIGURE 7:**
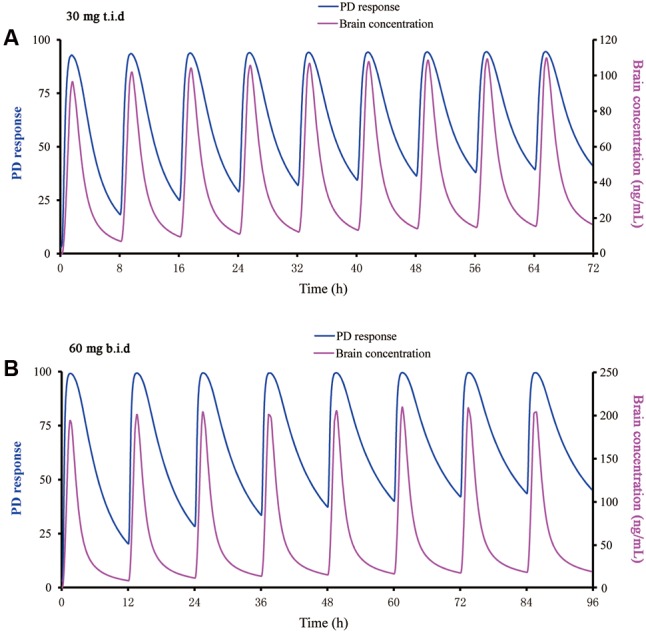
Predicted PD response using a direct sigmoidal *E*_max_ model and brain concentration versus time profile for buagafuran at 30 mg t.i.d. **(A)** and 60 mg b.i.d. **(B)** in man.

Using the PBPK/PD approach, various dosing regimens of buagafuran were simulated in human with body weight of 70 or 100 kg. It was found no significant effect of body weight on the exposure of buagafuran. A 30 mg t.i.d. dosing regimen of buagafuran appeared to reach *EC*_50_ for most of the day at steady-state (**Figure 7A**). PD response was not significantly increased with elevated brain concentration when the doses were higher than 30 mg. The dosing regimen of 30 mg t.i.d might obtain relatively satisfactory clinical response with low brain concentration. Therefore, 30 mg t.i.d. was considered the potentially effective dose for human.

## Discussion

During the preclinical study, the metabolism of buagafuran *in vitro* was studied with rat liver microsomes. The major metabolic pathway of buagafuran was hydroxylation and carbonylation. Eight main metabolites were identified using MS and NMR. Five of these metabolites were found in rat brain after oral dosing (Zhang, 1997). When buagafuran was incubated with human liver microsomes, similar metabolites were formed (Li et al., 2001; Li, 2010). And only one hydroxylation metabolite can be identified in human plasma after oral administration of buagafuran (Yang et al., 2013). In-house studies have shown that the metabolites of buagafuran have weak or negligible anxiolytic activity in animal studies. Therefore, the anxiolytic activity of buagafuran is mainly produced by itself.

The EPM model is one of the most widely employed behavioral tests for anxiety. This model, validated pharmacologically, physiologically and behaviorally, has been applied in the drug development including the benzodiazepines, ondansetron, barbituates, prazosin and clonidine (Cryan and Sweeney, 2011; Garg et al., 2011). The current human data are consistent with results from the rat EPM models in terms of anxiolytic effects. For example, the dosage of diazepam for anxiety is 2–10 mg 2 to 4 times a day. For a 60 kg person, the corresponding dose should be 0.06–0.67 mg/kg. The equivalent dose for rat is obtained using the formula as follows: Animalequivalent dose(mg/kg) = Human dose (mg/kg)× HumanKm÷AnimalKm. For a 60 kg adult (body surface area 1.6 m^2^), Km is 37. For a 150 g rat (body surface area 0.025 m^2^), Km is 6. Therefore, the rat dose should be 0.37–4.2 mg/kg. Consistently, diazepam can increase the time spent on the end sections of open arms at 0.5–10 mg/kg (Yasumatsu et al., 1994). Buagafuran exhibits significant anxiolytic activity in EPM model with an inverted U-shaped dose response. The results are confirmed by the social interaction test (Zhang et al., 2004). Inverted U-shaped dose response is a representative characteristic of common anxiolytics, such as benzodiazepines and buspirone (Jenck et al., 1997). In lower doses, benzodiazepines simply reduce anxiety, while in higher doses they act like sedatives and may promote sleep. Although there are several literatures on the inverted U-shaped dose response of anxiolytic agents in animal, few literatures can be found about the dose–response relationship in human. However, a literature compared the anxiolytic effects of two dosages of bromazepam (Harris and Kay, 1984). Two regimens showed significant improvement and the higher dose was no more efficacious. The most common adverse reaction volunteered was drowsiness and the occurrence of drowsiness was higher in the high dose group. Consequently, there is a strong possibility that anxiolytic agents exhibit the inverted U-shaped dose response in human. It is reported that Phase II trial success rate for experimental drugs was below 20% between 2009 and 2011, and the majority of the failures attributed to a short of efficacy (Arrowsmith and Miller, 2013). Given the U-shaped dose–response relationships of anxiolytics, it is important to predict a potentially effective dose of buagafuran for Phase II trial because too high a dose can hide its pharmacological effect.

Generally speaking, the no observed adverse effect level (NOAEL) method is commonly applied to predict the first-in-human dose based on pre-clinical data. NOAEL is an approach using simple allometry for extrapolation of animal PK parameters to the human situation on the basis of body surface area. This conservative approach is easy and practical with a good safety record. The premise of this method is the drug exhibits similar PK and PD response in two species. If this assumption is invalid, NOAEL method always underestimates the effective dose (Sharma and McNeill, 2009).

The PBPK modeling is an alternative to allometric scaling approach. Differ from compartmental model, PBPK model allows extrapolation for dose, species, or route based on real physiological data. Certainly, the development of PBPK models requires a large number of experiment data and deep understanding of target compound PK features. Recently, its application in dose selection is attracting a growing interest (Wagner et al., 2015). Compared with the data from allometric scaling approach, the prediction of PK parameters from PBPK model was more accurate in a retrospective analysis of 19 compounds carried out by Hoffmann-La Roche (Jones et al., 2006).

Buagafuran, an oily liquid at room temperature, is a Class II drug in biopharmaceutical classification system (BCS). Due to its physical-chemical features of the compound, it was very difficult to obtain many drug parameters (log P, particle density, etc). Therefore, the parameters of this compound listed in **Table 1** were mostly estimated by software. However, we measured the important PK parameters, such as apparent permeability of Caco-2 cells, F_Up_ and *CL*_int_. In PBPK modeling, CL is a key parameter to simulate PK profiles. The most accurate PBPK model input of CL should be obtained following intravenous administration. Due to the limitation of solubility, we did not conduct the PK description of buagafuran after intravenous injection in rat or human. As an alternative way, liver microsomes incubation could be used for CL estimation. However, in most cases, the predicted *CL* value was lower than the observed CL (Jones et al., 2011). In our study, in order to more accurately simulate the oral plasma concentration-time profiles of different doses of buagafuran, the refined instead of predicted *CL* value was used as input. Indeed, the predicted *CL* values (28.0 mL/min/kg for rat and 7.7 mL/min/kg for human) of buagafuran based on liver microsomes were lower than the refined values (36.8 mL/min/kg for rat and 11.7 mL/min/kg for human) optimized by observed PK data. Except for oxidative metabolites, glucuronide and sulfate conjugates of buagafuran have been reported in rats (Zhang, 1997). Therefore, ignoring the conjugation metabolism of buagafuran in microsomal system is one of the important reasons that cause the underestimated CL. On the other hand, since buagafuran undergoes extensive metabolism, it is speculated that the extrahepatic organs maybe contribute to the elimination of buagafuran. That systemic clearance was substituted by hepatic clearance maybe another important reason for the underestimation of the CL. In our study, the estimate CL from the microsome study was used to provide the basis for our simulation. Then, by constantly adjusting the CL to make the fitting curve consistent with the measured PK curve of different doses, the corrected CL is more accurate and can be used for predictions of other oral dosing. For compounds that microsomal metabolism cannot completely represent the *in vivo* elimination, if there is no measured PK data to improve the input CL, it would be impossible to obtain accurate prediction. In the present study, the input *CL*_hep_ values were refined by comparison of simulation with observed data, PK profiles of buagafuran in rats and in human were simulated well. In addition, the constants C1 and C2 value for Opt logD Model SA/V of the ASF model are important parameters relevant to the prediction of drug absorption. The constants C1 and C2 provide a convenient way to scale the ASFs to account for factors such as active transport and physiological changes in the small intestine. Utilizing an oral PK dataset of target drug, the ASF model can be optimized by changing constants C1 and C2. For example, Zhang et al. (2011) optimized the constants C1 and C2 to obtain optimized ASF values which were 10 times the Opt logD Model generated values, demonstrating a fast uptake of carbamazepine in the small intestine. Whereas the adjusted ASF value below the default value means that the efflux transporters may have an effect on the absorption of target drug (Jelena, 2013). In our study, the constants C2–C4 used the default values, while the constants C1 for rat and human were adjusted from 0.8805 to 0.6805 and 0.8005 to best fit the observed plasma concentration of buagafuran. The resultant ASF values for rat and human were about 63 and 83% of the default GastroPlus^TM^ values, which indicated that efflux transporters may affect the absorption of buagafuran. This assumption was supported by the results of Li, who found that buagafuran is a substrate of the transporter P-gp (Li, 2010).

Physiologically based pharmacokinetic model could not explain PD differences. However, it is helpful to optimize the dose estimate when integrating the PBPK model with PD modeling (Toutain, 2002). In our study, a PBPK/PD model was established to predict the therapeutic dose of buagafuran in human. The buagafuran rat PBPK model was developed using concentration data acquired from two dose levels. The PBPK model sufficiently depicted the plasma and brain concentration-time curves in rat model following administration of buagafuran. The PK/PD model was established using data at the dose on the plateau of the effectiveness dose-response curve. Although plasma PK properties have been successfully related to pharmacological effects in many cases and there is no delay for the distribution of buagafuran to brain, plasma concentration would not be a sufficient surrogate end point in PK/PD modeling. The concentration of drug in tissue is associated with physiological factors, such as blood flow and tissue volume, which contributes a complex relationship of drug plasma concentration with its tissue concentration in different species. Accordingly, it is perfect to develop a PK/PD model by linking the drug responses with the target site drug levels. Based on the ability of PBPK models in predicting tissue exposure to a drug, a direct-link PK/PD model describing the PD response with the brain concentration profile of buagafuran in the rat had been developed.

According to the results of PK-PD model simulation by GastroPlus software, Sigmoid *E*_max_ model was selected as the best-fitting model for PD response of buagafuran mainly based on the values of *R*^2^ and Akaike criterion. Sigmoid *E*_max_ model describing non-linear concentration–effect (C–E) relationships is a classic and commonly used PD model. This model is characterized by a sigmoidal shape and the Hill coefficient depicts the steepness of the profile. For buagafuran, the rat data provided appropriate coverage of the *EC*_50_ region. A relatively steeper sigmoid C–E curve was obtained with the Hill coefficient of 2.5.

While interspecies differences in pharmacological intensity and duration of action occur, PK/PD modeling remains a powerful approach to improve the translational application of preclinical studies to clinical studies. It has been reported that reasonable agreement of PD response of drugs (both macromolecule and small molecule) was found between animals and humans (Zuideveld et al., 2007; Mager et al., 2009; Kagan et al., 2010; Chang et al., 2011; Yamazaki, 2013). Mager et al. (2009) successfully scaled PD responses of recombinant human erythropoietin using a rat PK/PD model to humans. Kagan developed a PK/PD model of type I interferons with good predictive performance (Kagan et al., 2010). Yamazaki demonstrated that the PK/PD modeling of an anticancer drug, crizotinib, could be applied to the translational pharmacology from preclinical toward clinical development (Yamazaki, 2013). Zuideveld et al. (2007) also scaled pre-clinical PD of two 5-HT_1A_ receptor agonists to man. Chang et al. (2011) predicted the human *K*_i_ of aκ-opioid receptor antagonist based on a rat PK/PD model. The predicted *K*_i_ was consistent with the simulation results of the human PK/PD model (Chang et al., 2011). PD data scaling is largely dependent on the capability to estimate and combine the basic processes of drug exposure (pharmacokinetics), drug effects (pharmacology), and interaction with physiological systems. Certainly, PBPK modeling facilitates scale-up of PD data from animals to humans.

The buagafuran human PBPK model was developed and validated using concentration data from three single-dose and a multiple-dose studies. The PBPK model adequately depicted the plasma concentration-time curves in human following administration of buagafuran. The primary uncertainty in a combined PK/PD model is the extension of the PD model from animal pharmacology to human. Therefore, the entire construction relies on the assumption for the pharmacological similarity between human and rat. In addition, other assumptions had to be made, such as the same brain penetration and the same unbound fraction in the brain. Keep these restrictions in mind, buagafuran at the clinical dose of 30 mg t.i.d. was estimated to reach *EC*_50_ for most time of the day with a steady-state PK.

In addition, balance between efficacy and side-effects should be concerned in the dose selection. The starting dose of buagafuran for the Phase I clinical trial was based on one-sixtieth of the lowest dose that produces drug-induced biochemistry abnormal or pathological alterations in rats and dogs. It was found that buagafuran could be tolerated in several oral single-dose studies (15–90 mg), or multiple-dose studies (60 or 120 mg, b.i.d) in phase I trials. Still, side effects among which drowsiness is the most common were observed in these studies (Zhang, 1997; Li et al., 2001). PK/PD modeling allows for the estimation of the time process of drug action caused by a particular dosing regimen. The buagafuran PBPK/PD modeling showed that PD response was not significantly increased with elevated brain concentration when the doses were higher than 30 mg. For example, compared with the dose of 30 mg t.i.d, buagafuran at the dose of 60 mg b.i.d just yielded similar PD response when the brain concentration increased by approximately 100% (**Figure 7**). Therefore, 30 mg t.i.d. was a theory-based clinical regimen with the potentially effective. Keeping in mind considerations concerning numerical identification of models following general lack of unicity and stability of solutions for minimum with restrictions problems, the results given by software were globally analyzed it concerns their likelihood from phenomenological point of view.

## Conclusion

A potentially effective dose (30 mg t.i.d.) for buagafuran in human was predicted by a PBPK/PD modeling method. The PBPK models for buagafuran in rat and human were validated by observed data. The PD model was extrapolated from rat to human on the assumption of the pharmacological similarity between human and rat. The approach employing PBPK/PD modeling allows the prediction under consideration of various dosing regimens and variability in parameters.

## Author Contributions

FY and BW performed research and wrote the paper. ZL and XX performed research and analyzed data. WW and DY participated in research design and contributed to the writing of the manuscript. LS and YL designed research.

## Conflict of Interest Statement

The authors declare that the research was conducted in the absence of any commercial or financial relationships that could be construed as a potential conflict of interest.

## Abbreviations

ACAT: advanced compartmental absorption and transit
ASF: absorption scale factor
AUC: area under the curve
CL: Clearance
EPM: elevated plus-maze
GI: gastrointestinal
HPLC: high-performance liquid chromatography
IS: internal standard
LC-MS/MS: liquid chromatography coupled with a tandem mass spectrometer
PBPK: physiologically based pharmacokinetic
PD: pharmacodynamic
PVP: polyvinylpyrrolidone
